# A Window to *Toxoplasma gondii* Egress

**DOI:** 10.3390/pathogens7030069

**Published:** 2018-08-14

**Authors:** Lucio Ayres Caldas, Wanderley de Souza

**Affiliations:** 1 Instituto de Biofísica Carlos Chagas Filho, Universidade Federal do Rio de Janeiro, Rio de Janeiro, RJ 21941-902, Brazil; wsouza@biof.ufrj.br; 2 Centro Nacional de Biologia Estrutural e Bioimagem (CENABIO), Cidade Universitária, Rio de Janeiro, RJ 21941-902, Brazil

**Keywords:** *toxoplasma gondii*, *toxoplasma* egress, electron microscopy

## Abstract

The *Toxoplasma gondii* cellular cycle has been widely studied in many lifecycle stages; however, the egress event still is poorly understood even though different types of molecules were shown to be involved. Assuming that there is no purpose or intentionality in biological phenomena, there is no such question as “Why does the parasite leaves the host cell”, but “Under what conditions and how?”. In this review we aimed to summarize current knowledge concerning *T. gondii* egress physiology (signalling pathways), structures, and route.

## 1. Toxoplasma and Toxoplasmosis

*T. gondii* is an obligate intracellular protozoan parasitic member of the Apicomplexa family that infects nearly one third of the human population and causes a disease of high prevalence known as toxoplasmosis [1]. Effective immunity ensures an asymptomatic course, while immunocompromised individuals can experience non-specific clinical signs or mononucleosis-like symptomatology, being at risk for lethal disease development. Although subclinical infection is the most prevalent form, primary infection during pregnancy can lead to abortion, and congenital transmission is associated with mental disturbance development and deafness in addition to other complications [2,3].

*T. gondii* displays zoonotic characteristics and presents an alternate life cycle that is divided into sexual and asexual replication. During the first stage, which take places in the intestine of felids (the parasite’s definitive host), the encysted zygotes (named oocysts) are excreted and undergo meiosis that produces sporozoites. Once ingested by an intermediate host (a human, for example), these parasites convert to the tachyzoite form, which is responsible for the acute form of toxoplasmosis. Besides carnivorism, inhalation of dust contaminated with oocysts, and organ transplantation, means of transmission such as blood transfusion were also reported [4]. Otherwise, this form can also differentiate into the bradyzoite stage, which persists for long periods of time in the host organism.

*T. gondii* is capable of infecting almost all warm-blooded and nucleated cells, and at least three clonal lineages (types I, II, and III) are currently known, which differ in virulence and epidemiological occurrence [5]. Attenuated tachyzoites are employed in sheep vaccinations, but none are currently effective for humans or other animals. Nowadays, a combination of pyrimethamine and sulfadiazine with leucovorin is the first-line treatment for this disease. Although alternate therapies are also available, less toxic compounds are needed to support prolonged treatment [6,7].

## 2. Tachyzoite’s Cellular Cycle

A group of glycosylphosphatidylinositol (GPI)-anchored surface antigens (SAGs) and SAG-related sequence proteins present on the tachyzoite’s surface have been suggested to initiate the first interaction with the host cell. Not only the abundance of these proteins, but also their distribution, seem to favour the initial stage of parasitic attachment to the host cell [8,9].

As an Apicomplexa member, this parasite displays a complex of organelles in its apical region (micronemes and rhoptries). These secrete a variety of molecules needed to access the host cell. This access is facilitated by gliding motility, which is provided by an actin/myosin motor and is known to be crucial for invasion and egress [10]. This sequential secretion relies on micronemes, rhoptries, and dense granules.

Micronemal proteins (MICs) display conserved adhesive domains that strengthen attachment, and their secretion occurs concurrently with the tachyzoite’s conoid extrusion. This tachyzoite’s apical region projection also agrees with the transfer of rhoptries proteins (ROPs). These proteins are mostly involved in the moving junction formation that selectively sorts the containing proteins of the nascent parasitophorous vacuole (PV) membrane. Dense granules proteins are exocytosed after *T. gondii* invasion. The parasite, sheltered within the non-fusogenic PV, alters this compartment’s physiology followed by that of the tachyzoite itself [11,12]. This contributes to establishment and maintenance of an optimal environment for the survival and growth of the tachyzoites until egress, a step about which most details are not known, occurs.

## 3. Egress Signalling

*T. gondii* invasion and egress (in addition to motility and microneme secretion) are both accompanied by calcium (Ca^2+^) fluxes [13]. Because of this, and also because of the sharing of a few morphological aspects, they were initially interpreted as analogous processes [14]. Recent studies, however, concerning these phenomena point to distinct kinds of interactions that pose the existence of multiple determinants that may or may not converge with the egress. Indeed, aside from events such as those triggered by immune response, the role of Ca^2+^ in *T. gondii* egress is unavoidable [15,16].

The Ca^2+^ required for egress can be provided either by parasitic stores such as the endoplasmic reticulum (ER), acidocalcisomes, and the inner membrane complex [17,18]. Ca^2+^ can also be imported to the PV through non-selective pores in the parasitophorous vacuole membrane (PVM) following accumulation in the tubuvesicular network (TVN) [18]. It is worth noting that Ca^2+^ entry into host cells occurred through a nifedipine-sensitive Ca^2+^ channel. Ca^2+^ concentrations in the infected cells could increase via histamine release or thapsigargin inhibition of the ER Ca^2+^-ATPase, for example, but parasites were unaffected and not able to egress [19]. However, following the discovery of ionophore properties by Pressman [20], the pioneering works of Endo et al. [21] with Ca^2+^ ionophores were fundamental for allowing *T. gondii* synchronized egress in infected cell cultures. Since then, the majority of studies on *T. gondii* egress were performed in this way, relying on the ionophore-induced egress (IIE) [22,23,24]. This allowed the development of new tools to study egress [25] and led to identification of molecules that respond to the Ca^2+^-triggered signalling cascade.

### 3.1. Micronemes

Since the parasitic distribution of microneme proteins during Ca^2+^ ionophore-induced stimuli mimics the same pattern observed in *T. gondii* attachment and penetration [26], the role of microneme’s discharge was conferred on parasite egress. Ca^2+^ release from intra-parasitic compartments (such as the ER) can also induce invasion-related events such as protein secretion and cytoskeletal rearrangement of the apical end of the parasite [26,27]. Nowadays, it is known that not only motility and conoid extrusion, but also microneme secretion, can be enhanced by Ca^2+^ influx [19]. Inositol-1,4,5-triphosphate (IP_3_) production via phosphatidylinositol phospholipase C, together with calcium mobilization, contributes to diacylglycerol (DAG) release. In turn, this can be converted to phosphatidic acid by DAG kinase 1, which mediates the secretion of micronemes [28]. As a consequence, disruption of the membrane protein, MIC2, leads to a significant delay in host cell egress. Recent studies conducted by Gras et al. [29] showed that parasites initiated a significantly delayed motility and were still attached to each other.

Since rhoptries act in conjunction with micronemes during the invasion process, their role in egress was also investigated. However, in several studies where rhoptry secretion was blocked, *T. gondii* invasion was inhibited, but not egress [30,31].

### 3.2. Dense Granules

Although the use of Ca^2+^ ionophores allowed the identification of many calcium effectors, the way these effectors are regulated and detected are largely unknown. A role on Ca^2+^ regulation was suggested for the abundant protein dense granule antigen (GRA)1, which was shown to bind Ca^2+^ [32]. GRA41 is another potential physiological Ca^2+^ regulator. This protein localizes within a recognized candidate for Ca^2+^ storage, the TVN. The TVN is also known to be crucial for *T. gondii* cellular cycle regulation inside the PV, and the absence of GRA41 results in altered morphology of this structure [33].

In addition, Pszenny et al. [34] showed that *Toxoplasma gondii* lecithin:cholesterol acyltransferase (TgLCAT) is another possible participant in calcium-independent *T. gondii* egress. This enzyme is stored in dense granules and uses phosphatidylcholine as a substrate to form lysophospholipids in the PVM. This causes an increase in PVM permeability and modifies the ionic PV environment. The role of TgLCAT should be considered in a scenario of cholesterol-saturated conditions in replicating tachyzoytes. Disruption of PVM by this enzyme’s metabolites could facilitate the exit of parasites in a “nutritionally exhausted habitat” [34].

### 3.3. Perforin-Like Proteins

Perforin-like proteins (PLPs) have previously been reported in bacteria (such as Listeria), which helps this prokaryote to escape from its vacuole after invading the host cell [35]. PLPs encoded in the genome of the phylum Apicomplexa were shown to cause rapid membrane disruption via pore formation (∼100 Å diameter), allowing tachyzoite egress from host cells. The microneme secretion of *T. gondii* perforin-like protein 1 (TgPLP1) is calcium-dependent. The description of PLPs in *T. gondii* and the mechanism by which it allows the parasite to cross the PVM were described by Kafsack et al. [36] and Kafsack & Carruthers [37]. Most PLPs interact in the form of monomers with receptors located on the target membrane followed by oligomerization and pore formation. Nevertheless, it is not yet clear if this TgPLP1 alone is capable of weakening the PVM and if this activity extends to the host cell organelles and plasma membrane. In view of the apparent contradiction involving PVM permeabilization factors, Pszenny et al. [34] proposed that TgLCAT’s Ca^2+^-independent and TgPLP1’s Ca^2+^-dependent activities could be synergistic.

### 3.4. Calpain

Cysteine proteases are presumed to contribute to PVM and host cell plasma membrane rupture, thus facilitating *T. gondii* egress. Host cell calpains are capable of cytoskeletal and plasma membrane remodelling. Chandramohanadas et al. [38] proposed that the result of the parasite’s perforins’ activity could provide the Ca^2+^ required for calpain activation.

### 3.5. Kinases

The Ca^2+^-dependent protein kinases (CDPKs) found in Apicomplexans are common in plants [39]. The *T. gondii* CDPK1 (TgCDPK1) was shown to be required for microneme exocytosis [40] and on the other hand, TgCDPK3 was shown to be crucial for egress. The plant-like Ca^2+^-dependent protein kinase TgCDPK3, located in the inner side of the plasma membrane, was shown to regulate egress via phosphorylation of the actinomyosin motor of Toxoplasma motility [41,42]. First, IIE was shown to have failed in TgCDPK3-deficient parasites because they could not permeabilize the PVM and showed no microneme secretion. Consequently, gliding motility could not be activated [43], indicating that TgCDPK3 was required for Ca^2+^-mediated signalling events in the host cell ionic typical conditions.

Interestingly, TgCDPK3 was not necessary for microneme secretion [44] but was shown to be crucial to Ca^2+^ ionophore-induced egress [42], suggesting a role in environmental change sensing, which could trigger signalling that culminates with the parasite exit from host cells [45]. It is worth noting that TgCDPK3 malfunction can result in resistance to ionophore-induced death (IID) during prolonged treatments with Ca^2+^ ionophore although the reasons are unknown [46]. Although TgCDPK3 activity was linked to a rapid parasite exit from the host cell due to the phosphorylation of proteins needed for tachyzoite motility, the kinase substrates remain unknown. In an effort to identify TgCDPK3-regulated signalling pathways, Treeck et al. [47] demonstrated that the role of this enzyme is not limited to *T. gondii* egress, also occurring upstream from TgCDPK1. Nevertheless, TgCDPK3 seems to play an important role in regulating calcium basal levels within the parasite but could not be the main kinase required for the motor complex components. Subsequent studies have shown that in addition to TgCDPK2 and TgCDPK3, TgCDPK3 phosphorylates the parasitic motor protein, myosin A, which was shown to be essential for tachyzoite motility and egress [41].

In addition, the cGMP-dependent protein kinase G (TgPKG) was described as another member of egress signalling, required for Ca^2+^-independent microneme secretion. Its effects on the PI(4,5)P2 supply for phosphoinositide-phospholipase C ensures downstream liberation of inosotide triphosphate (IP_3_) and diacylglycerol (DAG) in addition to Ca^2+^ release from internal stores or ER [44,48]. Downstream from (or in conjunction with) TgDPKs that controls micronemes secretion, an apical complex lysine (K) methyltransferase (AKMT), was suggested as a parasitic motility regulator. AKMT could then trigger the parasite’s motile state via protein lysine methylation, thus contributing to the glideossome machinery activation [49].

Recently, Uboldi et al. [50] reported that the cAMP-dependent protein kinase A (PKA) negatively regulates egress speed, interferes with PV acidification, and decreases cytosolic Ca^+2^ signalling. Genetic down-regulation of a PKA subunit, PKAc, triggers premature *T. gondii* egress, suggesting that the holoenzyme’s activity is required to prevent egress in the presence of environmental acidification [51].

While other kinases (tyrosine kinase, protein kinase C, and phosphatidylinositol 3-kinase) inhibitors were shown to block *T. gondii* egress in early infective stages, this effect was less pronounced when the treatment was performed at times compatible with the natural egress [52,53].

### 3.6. Abscisic Acid Response

Abscisic acid (ABA), which is presumably synthetized in the apicoplast, triggers the production of cyclic ADP-ribose, leading to Ca^+2^ release. The main candidate for this Ca^+2^ release is the ER, and this activity culminates with microneme secretion and parasitic motility activation [54]. ABA accumulation between developing parasites may then act as a quorum sensing system since an increase in ABA levels was reported just prior to tachyzoites exit from host cells while ABA levels remain low during tachyzoite replication. Interestingly, blocking ABA leads to egress inhibition and also to bradyzoite development, indicating an important role for ABA in *T. gondii* differentiation [55].

### 3.7. Potassium Fluxes and pH

Another presumed quorum-sense system may operate via potassium (K^+^). While higher K^+^ concentrations preserves a non-motile parasitic state, lower K^+^ levels activate parasite motility via a parasitic phospholipase C and intraparasitic Ca^2+^ fluxes [15]. This egress model relies on the parasite’s ability to perceive environmental changes, then escape from an endangered host cell. Thus, treatment of *T. gondii* infected cells with the K^+^ ionophore, nigericin, which induces K^+^ efflux and changes host cell pH, successfully induced *T. gondii* egress [56]. By promoting host cell plasma membrane permeabilization, this process is then supposed to mimic an upstream event of intraparasitic Ca^+2^ fluxes. Although several Ca^+2^ responsive proteins have been identified, the way in which parasites sense the differences in K^+^ levels remains unidentified.

PV acidification prior to perforin secretion and *T. gondii* egress was the scenario proposed by Roiko et al. [57] after demonstrating that this decrease in pH was associated with parasitic motility enhancement and protein secretion. Since a low pH (pH 5.9–6.4) acting as the primary trigger in a high K^+^ environment could overcome K^+^ inhibition of microneme secretion. The PLP1 binding to the target membrane could be pH-dependent, and this model proposes that acidification would be sufficient to trigger tachyzoite egress.

### 3.8. External and Inflammatory Factors Triggering Cells

Unlike the egress observed under in vitro conditions, those that occur in inflammatory cells in vivo tend to be abbreviated [58]. During host immune responses, natural killer (NK) cells can trigger apoptosis in infected cells, leading to rapid parasite escape. These egressed parasites were also capable of infecting interacting natural killer (NK) cells [59]. In this case, the parasites would not only abandon the endangered cell but would also immediately invade another permissible host, incidentally assuring their propagation [58].

During host immune responses against *T. gondii*, interleukin (IL)-12 activates NK cells to produce gamma interferon (IFN-γ), leading to type I cluster of differentiation (CD)4^+^ and 8^+^ T-cell proliferation and production of more IFN-γ. IFN-γ appears to be the most important cytokine involved in protection against *T. gondii*. In astrocytes, *T. gondii* inhibition was previously shown to occur through an IFN-γ-inducible GTP-binding protein (IGTP-dependent mechanism). In murine astrocytes and macrophages, host cell ER derived-inducible IGTP could induce PV disruption just prior to tachyzoites motility detection [60]. In human foreskin fibroblasts (HFFs), IFN-γ also induced parasite egress, independent of tryptophan or iron deprivation [61].

IFN-γ further activates the production of nitric oxide (NO) or tumor necrosis factor (TNF) on *T. gondii*-infected macrophages, which helped to control the parasite replication. Thus, NO treatment was proven to successfully trigger *T. gondii* egress from infected immune (peritoneal macrophages) and non-immune cells (HFFs) via parasitic Ca^+2^ increase and motility [62,63].

Infected HFF cells also had their tachyzoites released upon treatment with TNF-α. This pro-inflammatory cytokine originating in the innate immune system was able to induce the early *T. gondii* egress at a TNF-α concentration of 10 ng/mL in a time-dependent manner. Besides intra-parasitic Ca^2+^ mobilization, parasitic motility and the host apoptosis pathway were shown to be crucial for this process, without affecting parasite virulence [64].

On the other hand, some artificial egress triggers were shown to be successful. For instance, ethanol-induced microneme secretion and Ca^+2^ increase have been shown to lead to early *T. gondii* egress [65]. The receptors involved in these reactions may belong to the inositol 1,4,5-triphosphate/ryanodine receptor superfamily [66]. In addition, Stommel et al. [67] used dithiothreitol (DTT) to activate nucleoside triphosphate hydrolase (NTPase) isoforms, causing parasite exit within 60 seconds. Borges-Pereira et al. [19] using ionomycin, reported high levels of parasitic and host cell Ca^+2^ increase even when the Ca^+2^ is absent from the buffer. The study indicates that both parasitic and host cell Ca^+2^ stores are sufficient to activate parasitic motility and egress.

## 4. Egress Structure and Route

The protruded conoid and release of secretory organelles are two characteristics of motile parasites, and these processes are accompanied by genetic expression rearrangements in response to environmental changes [68]. The protrusion of conoid is triggered by calcium stimuli and is believed to occur during *T. gondii* egress; however, it can be inhibited without affecting parasite motility [69,70].

Parasitic gliding motility results from the orchestrated action of a variety of players that form the so-called glideosome. This parasitic cytoskeletal-attached machinery situated between the plasma and internal membrane complexes interact with external substrates, thus propelling the tachyzoite forward. The growing importance of the glideosome for tachyzoite egress was recently reinforced by studies that approached the key effector molecules of its actinomyosin motor even though the signalling pathways that coordinate this activity are not yet well understood [10,71]. This mechanical force may be crucial, in conjunction with the players cited above, to ensure PVM and host plasma membrane crossing.

Melzer et al. [60] proposed that fusion performed by the host cell ER and PV as observed by electron and laser microscopic methods could play a role in PV disruption via Ca^+2^ discharge into the PV. On the other hand, in studies recently performed by Periz et al. [72], it was reported that an F-actin network connected the intravacuolar parasites to the PVM, the TVN, and the residual body. Ca^2+^ ionophore treatment resulted in disruption of the actin’s network, indicating another required step prior to PV exiting.

**Figure 1:**
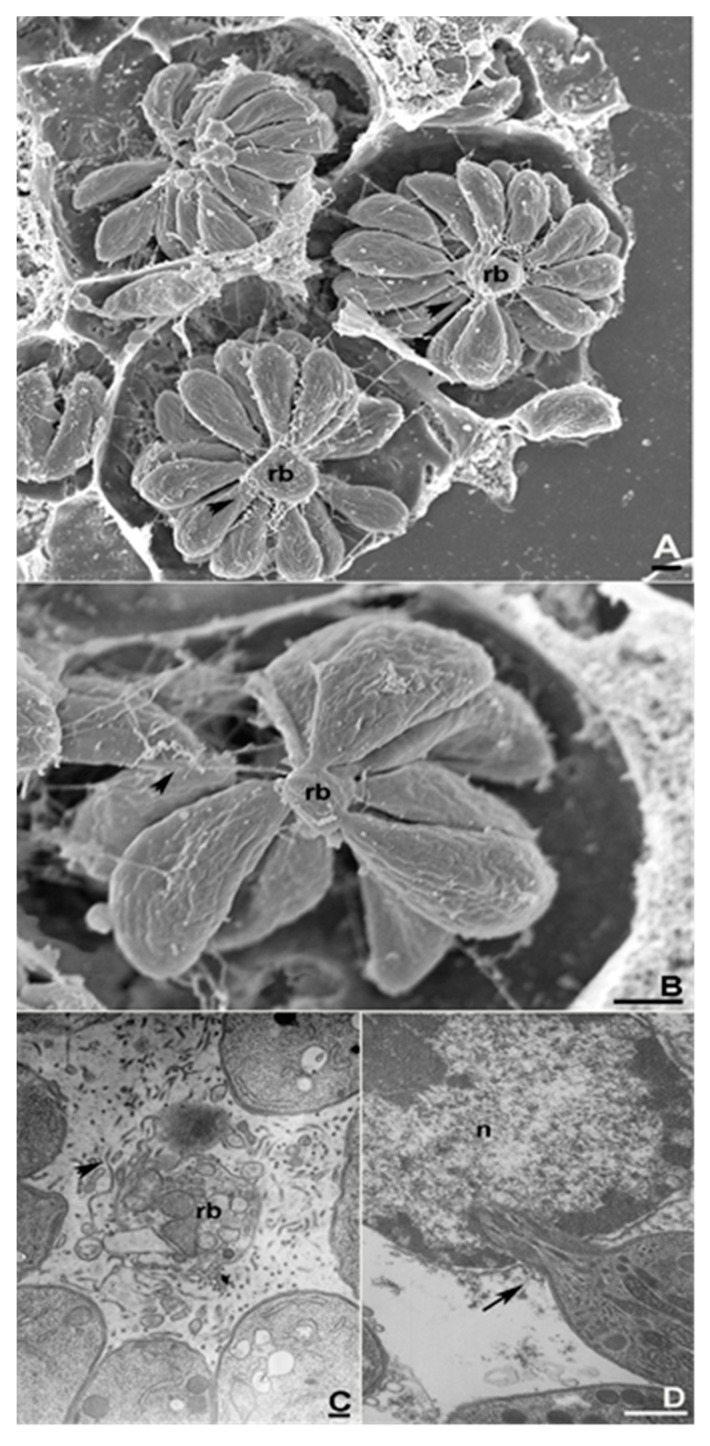
Electron microscopy of the early events on *T. gondii* egress from the host cell. (**A**) Scanning electron microscopy of three rosette-containing PVs. Arrowheads indicate the tubuvesicular network, which is stretched between the parasite and the residual body (**rb**) in (**B**), representing the first movements of the egressing tachyzoites. (**C**) Transmission electron microscopy of rosette disaggregation reveals the disorganisation of the tubuvesicular network (arrowhead) and the residual body. (**D**) Once in the host cell cytoplasm, tachyzoites can enter the host cell nucleus (arrow).

**Figure 2:**
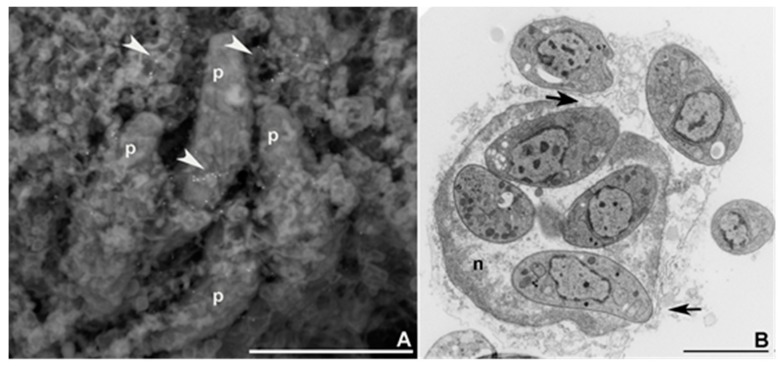
Electron microscopy of *T. gondii* egress from the PV and the host cell nucleus. (**A**) Backscattering electrons (scanning electron microscopy) showing gold-labelled tubulin (arrowheads) acting as a substrate during parasite (p) egress from the PV. (**B**) Transmission electron microscopy of tachyzoites egressing from the host cell nucleus.

After detachment from the TVN (Figure 1A,B), rosette disassembly, can occur, (Figure 1C) and the parasites can eventually push the PVM [24] toward the host nucleus direction (Figure 1D). Subsequent active disruption of PVM is then required for its crossing and subsequent gliding through the tubulin network around the PV (Figure 2A). Escape from the host cell nucleus was also documented (Figure 2B). Egress visualisation using electron microscopy revealed that the parasites carrying ER profiles in addition to PVM remnants showed adhered of the PVM to their apical portions [24]. This results from their apparently random route within the host cell cytosol, resulting in progressive clearance from these “barriers” and eventual ingress into the host cell nucleus [24,73].

**Figure 3:**
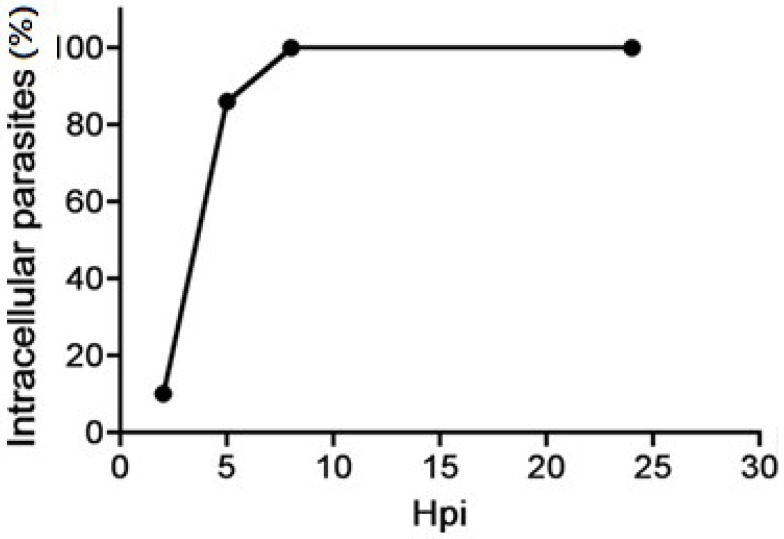
Reinfection assay of tachyzoites artificially egressed from host cells. Parasites egressed until 7 hpi from the host cell under calcium ionophore stimuli were unable to present an efficient infection.

Individual vacuoles observed at 24 h post-infection (hpi) could result from reinfection episodes that are performed by egressed tachyzoites in the cell culture. The presence of individual and collective vacuoles in the same cell suggests that an infected cell is still permissive to *T. gondii* infection. This poses a limitation on the speed of infection when compared to other obligate intracellular pathogens that tend to infect virgin cells [74]. Previous studies have demonstrated that these individually confined tachyzoytes can leave (at least after artificial stimuli) the host cell earlier in infection, but they become unable to establish a new cell infection [52]. We are then tempted to claim that a successful tachyzoite reinfection requires a kind of maturation process inside the PV. Studies performed with the monkey kidney epithelial cell line, LLCMK2, revealed that prematurely egressed tachyzoites were only ready to establish an efficient infection after at least 7 h within the PV (Figure 3). This phenomenon should be considered under the light of the studies on the immune response against *T. gondii* that results in premature egress [58,60,61]. Melzer et al. [60], for example, reported that IFN-γ induced egress from murine astrocytes at 6 hpi caused parasites to fail to undergo reinvasion. On the other hand, tachyzoites egressed from LLCMK2 cells after Ca^2+^ ionophore treatment at 2 hpi were able to invade, but not to infect, permissible cells [52].

Some authors have suggested that host cell cytoskeletal proteins can be cleaved by calpain at the host cell plasma membrane as a consequence of Ca^2+^ signalling-triggered egress [38]. The direct contact of the egressing tachyzoite with the host cell microtubules and microfilaments suggest a role for this event as a substrate for the parasite gliding. However, since egress was reported to occur predominantly in the loci of injured host cell actin, the host cell cytoskeleton can be seen as having a dual role in this route [53,75].

While drifting in the host cell cytosol the tachyzoites could be observed to assume an hourglass shape due to the obstacles faced in these ambient conditions (Figure 1D). The hourglass shape was also observed within egressing parasites while crossing the PVM and host cell plasma membrane.

Dogga et al. [76] showed that among seven aspartyl proteases (ASPs) found in *T. gondii* tachyzoites, ASP3, an endosomal-like compartment resident protease, is intimately associated with rhoptry discharge. Acting as a maturase for the parasitic apical organelles, this enzyme’s depletion results in egress impairment, possibly preventing efficient the rhoptry discharge that is considered necessary for host cell plasma membrane lysis.

**Figure 4:**
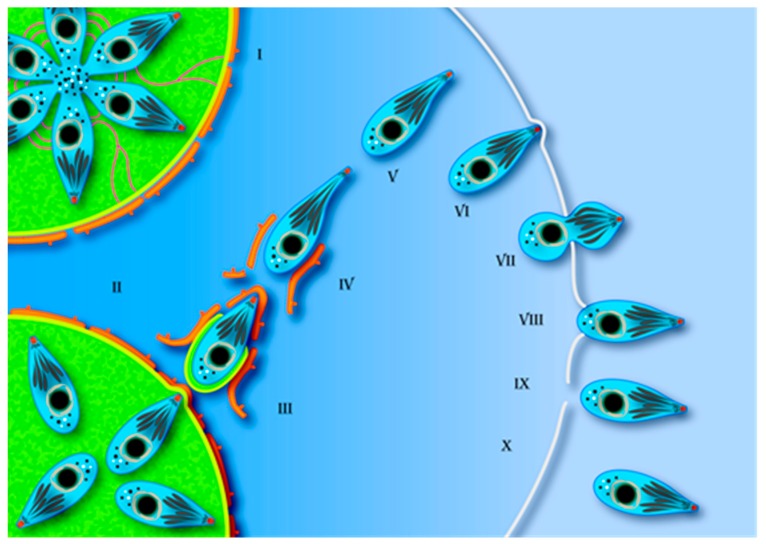
Schematic drawing of *T. gondii*’s tachyzoite egress from the host cell. (**I**) After many rounds of endodiogeny within the PV, the tachyzoites assume the rosette conformation; (**II**) Under determined stimuli that includes the participation of the calcium ion, the parasites become motile and the rosette conformation is disassembled. PV membrane pushing and permeabilization allow the arrival in the cytosol, where the parasites drag PV membrane and host cell ER profiles (red lines) (**III**); (**IV**) Stretching or constriction of the tachyzoite’s cell body can occur as the parasite moves through the cytosol contents and (**V**) looses the membrane remnants towards the extracellular domain (**VI**); (**VII**) When crossing the host cell plasma membrane (with the aim of perforins secretion?), the tachyzoite assumes an hourglass shape; (**VIII**) At the final of this process, the host cell plasma membrane may reseal (**IX**) or lysis can occur (**X**), as in the case of collective egress.

It’s also of note that host cell lyses, together with fast growth, is usually associated with tachyzoite infection, but tachyzoite budding from host cell plasma membrane was previously shown to occur under Ca^2+^ ionophore-induced and “natural” egress. Structures that resemble a remnant of individual egress have been recently described [75], but it remains unclear if they are a clue to the exit of individual tachyzoites. In the affirmative case, the host cell membrane should be rapidly fixed in order to avoid extracellular content invasion and intracellular leakage [24,75]. The above-referred steps of *T. gondii* egress structural details are summarized in our proposed model (Figure 4).

## 5. Perspectives

Despite being a prototype for the study of obligated intracellular protozoan parasites, the mechanisms and signalling pathways that lead to *T. gondii* egress are increasingly intricate and at the same time reveal new avenues to be explored. Intriguing questions still plague the debate, including the Ca^2+^ sources, the existence of quorum sensing mechanisms, the factors involved in unsuccessful infections from prematurely egressed parasites, the conditions that determine a hierarchy of egress contributors, and others. These unresolved subjects, however, have been shown to stimulate the design of more complex assays needed in this field. In view of the large number of players that contribute to *T. gondii* egress, we are just beginning to assemble this intriguing puzzle, relying on described parasite/host cell interaction factors and signalling molecules that, with the unavoidable command of Ca^+2^, converge at the exit event.
